# Construction and validation of a nomogram based on clinical indicators for 28-day composite poor prognosis prediction in severe sepsis

**DOI:** 10.3389/fpubh.2026.1709504

**Published:** 2026-02-10

**Authors:** Xinfu Chen, Zhuo Chen, Xuebing Liu, Peng Guo, Ruihua Shi

**Affiliations:** 1 Department of Gastroenterology, Medical School, Southeast University Affiliated Zhongda Hospital, Nanjing, Jiangsu, China; 2 Department of Gastroenterology, Xuzhou Municipal Hospital Affiliated to Xuzhou Medical University, Xuzhou, Jiangsu, China

**Keywords:** biomarkers, intestinal barrier dysfunction, prediction model, prognosis, sepsis

## Abstract

**Objective:**

To develop and internally validate an individualized nomogram integrating intestinal barrier-specific biomarkers and systemic clinical indicators to help assess intestinal barrier function and provide a reference for prognosis prediction in patients with severe sepsis.

**Methods:**

Three hundred fifty-two patients with severe sepsis admitted between January 2022 and December 2024 were continuously enrolled and randomly divided into training (*n* = 246) and validation (*n* = 106) sets. Plasma samples and clinical data—including demographics, injury assessments, and initial laboratory indicators—were collected. Prognosis-related variables were identified via univariate analysis. LASSO regression was used for variable selection, and multivariate logistic regression identified independent predictors of poor prognosis. Model performance was evaluated using receiver operating characteristic (ROC) curves, calibration plots, and decision curve analysis (DCA) in both training and validation sets

**Results:**

Baseline characteristics did not differ significantly between sets (all *P* > 0.05). Multivariate analysis identified admission SOFA score, intestinal fatty acid-binding protein, D-lactate, procalcitonin, and blood lactate as independent risk factors for poor prognosis (all *P* < 0.05). The nomogram demonstrated good calibration and fit, with C-indexes of 0.771 (training) and 0.641 (validation), mean absolute errors of 0.026 and 0.043, and non-significant Hosmer-Lemeshow test results (*P* = 0.423 and *P* = 0.496, respectively). The AUCs were 0.771 (95% CI: 0.698–0.845) and 0.641 (95% CI: 0.512–0.770), with sensitivities of 0.672 and 0.462, and specificities of 0.804 and 0.800.

**Conclusion:**

The constructed nomogram, incorporating intestinal barrier biomarkers and systemic clinical indicators, can help assess intestinal barrier-related risk and provide a reference for predicting adverse outcomes in severe sepsis. It offers a valuable decision-support tool for early goal-directed intervention and demonstrates significant clinical translational potential.

## Introduction

Sepsis is a life-threatening organ dysfunction caused by a dysregulated host response to infection. It is one of the leading causes of death among patients in the Intensive Care Unit (ICU) globally. Its incidence is still increasing year by year, imposing a heavy burden on the healthcare system (1). The mortality rate of severe sepsis and septic shock was as high as 30%−50% (2). Its pathophysiological mechanism has not been fully elucidated yet.

In the complex pathophysiological network of sepsis, the intestinal mucosal barrier, composed of mechanical, chemical, immune, and microbial barriers, is an important line of defense against the invasion of bacteria and toxins from the intestine into the systemic circulation (3). In the state of sepsis, due to factors such as systemic hypo perfusion, inflammatory mediator storm, oxidative stress, and immune dysfunction, the structural integrity of the intestinal barrier is severely damaged, manifested as apoptosis and necrosis of intestinal epithelial cells, destruction of tight-junction proteins, and a significant increase in intestinal permeability (4). These series of changes further exacerbate the Systemic Inflammatory Response Syndrome (SIRS) and Compensatory Anti-Inflammatory Response Syndrome (CARS), thereby triggering or aggravating the Multiple Organ Dysfunction Syndrome (MODS), forming a vicious cycle that is difficult to break (5, 6). Therefore, early assessment and protection of intestinal barrier function are considered one of the key strategies to improve the prognosis of sepsis patients.

In recent years, a series of plasma biomarkers have shed light on the non-invasive assessment of intestinal barrier function. Intestinal Fatty Acid Binding Protein (I-FABP) is a specific protein present in the cytoplasm of mature intestinal epithelial cells. It is rapidly released into the blood during cell ischemia and necrosis and is an early and sensitive indicator of intestinal epithelial cell damage (7). D-lactate is a metabolite of intestinal bacterial fermentation. Mammalian cells cannot produce it in large quantities. An increase in its plasma level directly reflects an increase in intestinal permeability and bacterial product translocation (8). Diamine oxidase (DAO) is mainly distributed in the upper cells of intestinal mucosal villi. After cell damage, its activity increases and is released into the blood, which can be used as an indicator of intestinal mucosal integrity and renewal rate (9). Although these biomarkers show great potential, the predictive value of a single indicator is limited, and a more powerful comprehensive prediction system needs to be constructed.

Based on the above background, this study intends to systematically detect the levels of intestinal barrier-specific biomarkers in the early stage of patients with severe sepsis and combine them with routine critical illness assessment indicators (10) to construct a visual and clinically convenient individualized prediction nomogram model.

## Materials and methods

### Study subjects

This study was a single-center retrospective observational study conducted at the ICU of Southeast University Affiliated Zhongda Hospital and Xuzhou Municipal Hospital Affiliated to Xuzhou Medical University. A total of 352 patients with complete I-FABP and D-lactate data (≈60–78% of the total admitted severe sepsis patients) were included by reviewing electronic medical records (EMR): eligible patients were screened based on the predefined inclusion and exclusion criteria, and all qualified patients during this period (January 2022–December 2024) were included consecutively to ensure the completeness of the cohort. Inclusion criteria: (1) Age ≥ 18 years, (2) Meeting the diagnostic criteria for sepsis in “Surviving Sepsis Campaign: International Guidelines 2021” (11) and the Sepsis-3 international consensus (12), that was, having suspected or confirmed infection, and the Sequential Organ Failure Assessment (SOFA) score increased by ≥ two points compared with the baseline, (3) Having serum samples available for the detection of I-FABP and D-lactate within 24 h of admission. Exclusion criteria: (1) Pregnant or lactating women, (2) Complicated with intestinal tumors (regardless of survival prognosis), (3) Complicated with advanced malignant tumors and expected survival < 3 months, (4) Suffering from severe chronic intestinal diseases (such as Crohn's disease, ulcerative colitis, short-bowel syndrome) or having a history of recent (< 3 months) intestinal surgery, (5) Missing or incomplete clinical data, (6) Patients who were discharged voluntarily or gave up treatment. Among the initially screened 371 patients, 19 (5.1%) were excluded due to missing key data (e.g., I-FABP, D-lactate, SOFA score), resulting in a final sample size of 352. The retrospective cohort of 352 patients was statistically split into a training set (*n* = 246) and a validation set (*n* = 106) at a 7:3 ratio using a random number generator (not prospective patient randomization). This study was approved by the Ethics Committee of Hospital. Written informed consent was obtained from all participants.

### Sample size estimation

The sample size of this study was estimated based on the general principles of developing predictive models. To ensure the stability and generalization ability of the model, the standard of event count per variable (EPV≥10) was followed. The main outcome of this study was 28-day composite poor prognosis (defined as dependence on mechanical ventilation, renal replacement therapy, or vasopressor support with failure to transfer out of the ICU). Based on preliminary data from our hospital, approximately 28–31% of critically ill sepsis patients admitted to the ICU will experience this composite poor prognosis, which is consistent with the observed incidence in this study (training set: 26.83%; validation set: 31.13%). In the final multivariate logistic regression model, it was planned to include approximately 8–10 predictor variables (including selected meaningful indicators and necessary clinical covariates). Therefore, the minimum number of events required (i.e., the number of “high-risk” patients) was: 10 (EPV) x 10 (variables) = 100 events. Based on a 50% event incidence rate, the total sample size required was estimated to be at least: 100 (events)/0.5 (incidence rate) = 200 patients. Considering that there may be about 20% of data missing or excluded during the research process (such as due to poor serum sample quality, incomplete clinical data, etc.), the sample size was increased by 20% on this basis, and the final target sample size was: 200/(1–0.2) = 250 patients. The three-year patient cohort (January 2022 to December 2024) to be included in this study exceeds this limit.

### Data collection

Patient data were collected through the hospital's Electronic Medical Record System (EMR): (1) Demographic data: age, gender, body mass index (BMI), (2) Underlying diseases and medical history: hypertension, diabetes, (3) Infection-related indicators: site of infection (such as lungs, abdomen, bloodstream, etc.), (4) Laboratory indicators within 24 h of admission: platelet count, creatinine, total bilirubin, C-Reactive Protein (CRP), Procalcitonin (PCT), blood lactate, Disease severity scores: SOFA score, Acute Physiology And Chronic Health Status Score II (APACHE II). The scores were independently calculated by two trained physicians, and differences were resolved through consensus. (6) Detection of intestinal barrier function biomarkers: The remaining serum samples collected within 24 h of admission and stored in a-80 °C freezer were thawed in a unified batch. The levels of I-FABP and D-lactate were detected using the Enzyme Linked Immuno Sorbent Assay (ELISA). All operations were strictly performed according to the kit instructions by researchers unaware of the patients' clinical outcomes. The coefficient of variation (CV) of the internal quality control products was < 10%.

### Outcome definition

With reference to “International Guidelines for the Management of Sepsis and Septic Shock (2021 Edition)” and “ESPEN Guidelines on Enteral Nutrition: Intensive Care (2019 Edition)”, the 28-day follow-up after admission to the ICU was set as the endpoint. The poor-prognosis group (defined as intestinal barrier dysfunction-related poor prognosis): Within 28 days after ICU admission, patients who still relied on mechanical ventilation, Renal Replacement Therapy (RRT), or vasoactive drugs to maintain vital signs and failed to be successfully transferred out of the ICU were considered to have poor-prognosis events. This outcome reflects persistent multi-organ dysfunction associated with intestinal barrier impairment and does not equate to mortality. The good-prognosis group (defined as relatively intact intestinal barrier function): Within 28 days after ICU admission, patients who were discharged from the hospital or remained alive without reliance on the above life-support measures were considered to have good-prognosis events. The good-prognosis group (i.e., the group with relatively intact intestinal barrier function): Patients who were discharged or still alive within 28 days were considered to have good-prognosis events.

### Statistical analysis

Statistical analysis was performed using SPSS software (version 26.0) and *R* software (version 4.2.3). Quantitative data were expressed as mean ± standard deviation (mean ± SD), and the *t*-test was used for comparisons between different groups. Qualitative data were presented as frequencies and percentages, and the χ^2^ (chi-square) test was used to evaluate the differences between groups. First, in the training set, all candidate variables (including demographics, clinical indicators, and biomarkers) were first subjected to Least Absolute Shrinkage and Selection Operator (LASSO) regression for variable compression (regardless of univariate significance) to avoid double-selection bias. Variables selected by LASSO (lambda.1se criterion) were then subjected to multivariate logistic regression to identify independent risk factors for 28-day composite poor prognosis (*P* < 0.05), and their odds ratios (OR) and 95% confidence intervals (CI) were calculated. Variance inflation factors (VIF) were calculated to exclude multicollinearity (VIF threshold < 10). For continuous predictors (I-FABP, D-lactate, PCT, blood lactate, SOFA score), the linearity assumption for the logit was tested via Box-Tidwell test, and no significant deviation was found (all *P* > 0.05), so no transformation or categorization was performed. Based on the finally determined independent risk factors, a nomogram model was constructed using the rms package. The operating characteristic curve (ROC) curve was plotted, and the areas under the curve (AUC) value was calculated. A model was considered to have good accuracy when the AUC value was between 0.7–0.9, and extremely high accuracy when the AUC value was >0.9. The calibration curve was plotted and evaluated using the Hosmer-Lemeshow goodness-of-fit test. The closer the calibration curve is to the 45-degree diagonal and the *P*-value of the H-L test >0.05, the better the consistency between the predicted probability of the model and the actual incidence. Decision curve analysis (DCA) was used to evaluate the clinical application value of the nomogram by calculating the net benefit at different threshold probabilities. A *P* value < 0.05 was considered statistically significant.

## Results

### Comparison of general data between patients in the training set and the validation set

A total of 352 patients were included in this study. Among the 246 patients in the training set, 180 cases (73.17%) were in the good-outcome group and 66 cases (26.83%) were in the poor-outcome group. Among the 106 patients in the validation set, 73 cases (68.87%) were in the good-outcome group and 33 cases (31.13%) were in the poor-outcome group. There were no statistically significant differences in the general data between the training set and the validation set (all *P* > 0.05) (Table 1).

**Table 1:** Comparison of general data between patients in the training set and the validation set.

**Indicators**	**Training set (*n* = 246)**	**Validation set (*n* = 106)**	***t/χ^2^***	***P***
Age (years)	65.40 ± 12.80	66.10 ± 11.90	0.481	0.631
Gender (male/female)	148/98	65/41	0.042	0.838
BMI (kg/m^2^)	23.80 ± 3.91	24.13 ± 3.72	0.737	0.462
Diabetes (yes/no)	68/178	25/81	0.627	0.428
Hypertension (yes/no)	115/131	55/51	0.783	0.376
APACHE II score	24.71 ± 6.49	25.23 ± 6.08	0.703	0.483
SOFA score at admission	9.81 ± 3.39	10.08 ± 3.17	0.699	0.485
I-FABP (pg/mL)	385.64 ± 215.73	401.33 ± 198.51	0.641	0.522
D-lactate (μg/mL)	45.21 ± 18.96	47.15 ± 7.59	1.018	0.309
DAO (U/L)	28.42 ± 10.29	29.60 ± 9.82	1.001	0.318
PCT (ng/mL)	35.67 ± 42.10	38.96 ± 45.33	0.657	0.512
CRP (mg/L)	185.60 ± 75.31	192.42 ± 70.81	0.793	0.428
Blood lactate (mmol/L)	3.82 ± 2.11	3.96 ± 2.03	0.578	0.564
Creatinine (μmol/L)	198.54 ± 105.70	205.37 ± 98.65	0.567	0.571
Total bilirubin (μmol/L)	45.67 ± 30.24	48.30 ± 28.91	0.758	0.449
Platelet count ( × 10^9^/L)	125.39 ± 75.61	118.92 ± 70.30	0.752	0.453

### Univariate analysis of influencing factors for poor prognosis in severe sepsis

Univariate analysis showed that, with the 28-day prognosis of severe sepsis in the training set as the gold standard, indicators such as the SOFA score at admission, I-FABP, D-lactate, PCT, and blood lactate had statistically significant differences between the good-prognosis group and the poor-prognosis group (all *P* < 0.05) (Table 2).

**Table 2:** Univariate analysis of influencing factors for poor prognosis in severe sepsis with intestinal barrier function.

**Indicators**	**Good-prognosis group (*n* = 180)**	**Poor-prognosis group (*n* = 66)**	***t/χ^2^***	***P***
Age (years)	65.13 ± 13.14	66.90 ± 11.70	0.963	0.337
Gender (male/female)	103/77	41/25	0.039	0.844
BMI (kg/m^2^)	23.74 ± 3.90	24.10 ± 3.70	0.650	0.516
Diabetes (yes/no)	58/122	15/51	2.086	0.149
Hypertension (yes/no)	80/100	35/31	1.430	0.232
APACHE II score	24.80 ± 6.67	25.97 ± 5.81	1.260	0.209
SOFA score at admission	9.67 ± 3.49	11.21 ± 3.04	3.170	0.002
I-FABP (pg/mL)	385.67 ± 215.74	464.03 ± 198.51	2.577	0.011
D-lactate (μg/mL)	45.17 ± 19.21	53.12 ± 7.43	3.270	0.001
DAO (U/L)	28.17 ± 10.14	30.11 ± 9.78	1.342	0.181
PCT (ng/mL)	34.79 ± 41.23	49.10 ± 47.23	2.318	0.021
CRP (mg/L)	184.76 ± 75.23	192.54 ± 70.69	0.730	0.466
Blood lactate (mmol/L)	3.73 ± 2.40	4.76 ± 2.14	3.067	0.002
Creatinine (μmol/L)	197.68 ± 105.42	206.49 ± 97.81	0.592	0.555
Total bilirubin (μmol/L)	45.18 ± 30.47	48.57 ± 28.96	0.783	0.434
Platelet count ( × 10^9^/L)	127.46 ± 75.31	117.89 ± 69.81	0.900	0.369

### Multivariate logistic regression analysis of influencing factors for poor prognosis in severe sepsis

Taking the prognosis of severe sepsis as the dependent variable (0 = good-prognosis group, 1 = poor-prognosis group), the indicators with statistical significance in the univariate analysis were included in the LASSO regression for variable screening (Supplementary Table 1). Variables were selected using the screening criterion of lambda.1se (Supplementary Figures 1, 2). The appropriate predictive variables were the SOFA score at admission, I-FABP, D-lactate, PCT, and blood lactate. These indicators were included in the multivariate Logistic regression analysis. The results showed that the SOFA score at admission, I-FABP, D-lactate, PCT, and blood lactate were independent risk factors for poor prognosis in patients with severe sepsis (all *P* < 0.05) (Table 3). In the regression model, the tolerance of each variable was > 0.1, the VIF was < 2, the condition index was < 30, and there was no situation where the variance proportion of multiple covariates under the same eigenvalue was >50%. Therefore, there was no collinearity among the covariates.

**Table 3:** Multivariate logistic regression analysis for determining the prognosis of severe sepsis.

**Indicators**	***B***	***SE***	***Wald***	***P***	***OR***	**95%CI**
SOFA score at admission	0.162	0.049	10.726	0.001	1.175	1.067–1.295
I-FABP	0.002	0.001	8.604	0.003	1.002	1.001–1.004
D-lactate	0.033	0.011	9.926	0.002	1.034	1.013–1.056
PCT	0.009	0.004	6.382	0.012	1.009	1.002–1.017
Blood lactate	0.199	0.069	8.300	0.004	1.220	1.066–1.397

### Development of the nomogram prediction model

**Figure 1:**
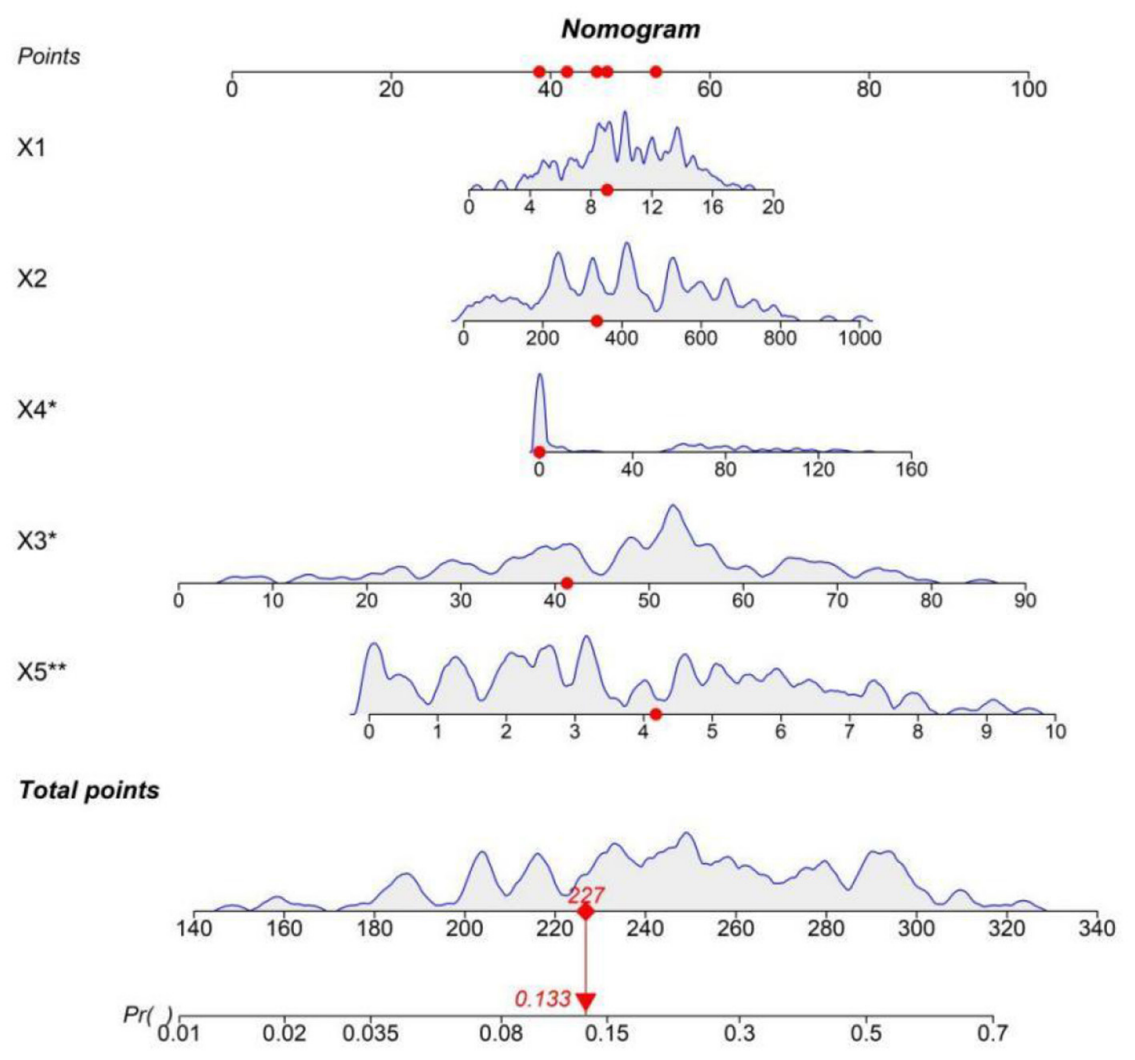
Nomogram prediction model for prognostic risk of severe sepsis.

Based on the results of the multivariate Logistic regression analysis, a nomogram prediction model for severe sepsis was constructed. In the constructed nomogram, each risk factor was assigned a specific scale segment. By accurately locating the actual values of each risk factor of the patient on the corresponding scale segment and then projecting vertically upwards, the score values corresponding to each risk factor could be obtained. Summing up these score values, the total score was obtained. The predicted probability value corresponding to the total score represented the individualized poor-prognosis probability of patients with severe sepsis (Figure 1).

### Evaluation and validation of the nomogram prediction model

**Figure 2:**
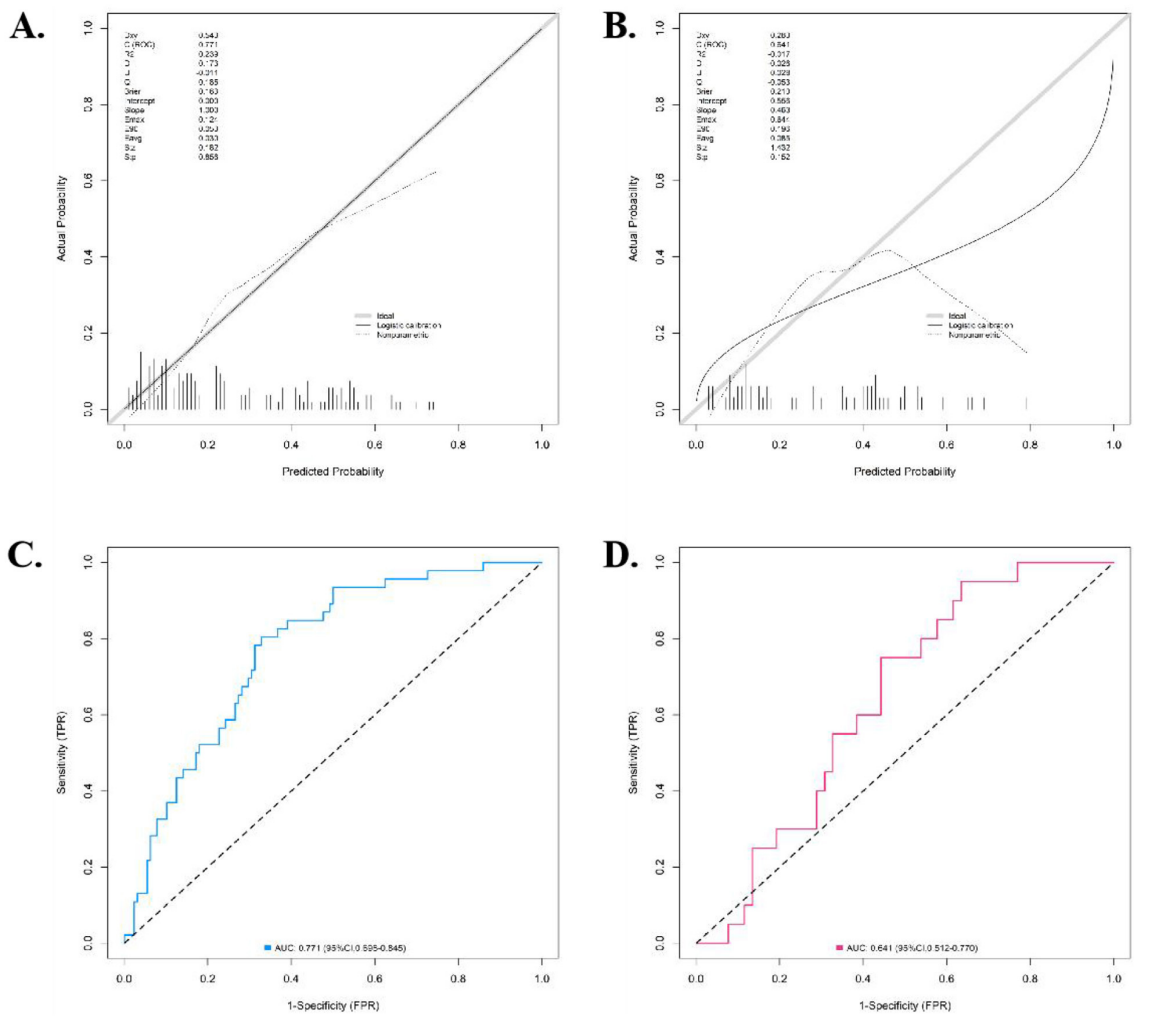
Calibration curve and ROC curve (**A**: Calibration curve of the training set, **B**: Calibration curve of the validation set, **C**: ROC curve of the training set, D: ROC curve of the validation set).

The nomogram model showed good calibration and goodness-of-fit between the predicted values and the actual values in both the training set and the validation set (the C-index was 0.771 and 0.641, respectively, the mean absolute errors of the agreement between the predicted values and the true values were 0.026 and 0.043, respectively, and the results of the Hosmer-Lemeshow test were χ^2^ = 8.104, *P* = 0.423 and χ^2^ = 7.382, *P* = 0.496, respectively). The ROC curves showed that the AUC for predicting 28-day composite poor prognosis of patients with severe sepsis by the nomogram model in the training set and the validation set were 0.771 (95% CI: 0.698–0.845) and 0.641 (95% CI: 0.512–0.770), respectively. The training set AUC reflected moderate-to-good discriminatory ability, and the validation set AUC reflected moderate discriminatory ability, indicating the model has acceptable predictive performance. The results showed that the model exhibited moderate-to-good predictive performance in the training dataset and moderate predictive performance in the validation dataset, with acceptable calibration and goodness-of-fit in both sets. The calibration curves and ROC curves were shown in Figure 2.

Bootstrapping validation with 1,000 repetitions, resulting in corrected C-indexes of 0.753 (training set) and 0.628 (validation set). Five-fold cross-validation, with a mean AUC of 0.732 (95% CI: 0.681–0.783) in the training set and 0.615 (95% CI: 0.502–0.728) in the validation set (Supplementary Table 1). These results confirmed the model's stability. Additionally, the EPV in the training set was 13.2 (66 poor-prognosis events/five predictors), which met the recommended threshold (EPV≥ 10) and further supported the model's stability.

### Decision curve analysis of the nomogram prediction model

The decision curve showed that when the threshold probability was approximately between 0.1–0.9, the decision of using the nomogram model constructed in this study to predict the risk differences caused by the influencing factors of the prognosis of patients with severe sepsis had more clinical benefits than the pre-operative decisions of considering all patients as damaged or all patients as undamaged (Supplementary Figure 3).

## Discussion

The main finding of this study is that, based on a prospective observational study, a nomogram prediction model integrating intestinal barrier-specific biomarkers (I-FABP, D-lactate) and systemic clinical indicators (SOFA score, blood lactate, PCT) was successfully developed and validated. This model can individually assess the 28-day adverse risk of patients with severe sepsis in the early stage after admission to the ICU, with moderate-to-good discriminatory ability in the training set (AUC = 0.771) and moderate discriminatory ability in the validation set (AUC = 0.641), providing a reference for clinical decision-making., providing a powerful decision-making aid for clinicians.

This study highlights the importance of intestinal barrier injury in the prognosis of sepsis: I-FABP (a highly specific surrogate for intestinal epithelial cell injury) and D-lactate (a classic indicator of increased intestinal permeability) were confirmed as independent predictors, while systemic indicators (SOFA score, blood lactate, PCT) supplemented the assessment of overall patient status. I-FABP is a highly specific marker of intestinal epithelial cell injury. Factor analysis confirmed that it is one of the strongest independent predictors (OR = 1.002), which is consistent with the conclusions of several previous studies (13, 14). The increase in its level directly reflects the ischemic necrosis of cells at the top of intestinal villi and is a sensitive indicator of intestinal hypo perfusion and injury. As a classic indicator reflecting the increase in intestinal permeability, the predictive value of D-lactate was also independently confirmed (OR = 1.034) (15). It is produced by intestinal bacteria, and an increase in its plasma concentration indicates the destruction of the physical barrier and the occurrence of bacterial/product translocation. These two markers jointly depict the functional status of the intestinal barrier from different perspectives (cell injury and permeability) (16). Their inclusion highlights the necessity of incorporating intestinal barrier assessment into the sepsis prognosis assessment system.

Meanwhile, the model also includes the cornerstone indicators reflecting the general condition. The SOFA score is the international gold standard for evaluating the degree of organ dysfunction and its close correlation with the adverse rate has been widely confirmed (17). The blood lactate level is a metabolic marker of tissue perfusion and cellular oxygenation disorders and is a core indicator for the resuscitation management of septic shock. Its predictive value is beyond doubt (18, 19). As a sensitive marker of the systemic inflammatory response, a continuously high level of PCT indicates poor infection control and an out-of-control inflammatory response (20, 21). By combining these systemic indicators with intestinal-specific indicators (22), the model provides a comprehensive and multi-dimensional assessment perspective from “local injury” to “systemic response”, which may be an important reason for its extremely high prediction accuracy.

Methodologically, this study strictly followed the research norms of clinical prediction models. A prospective design was adopted, the inclusion and exclusion criteria were clearly defined, and robust statistical methods were used. First, LASSO regression was used for the initial screening of variables, effectively avoiding the problems of overfitting and multicollinearity. Then, multivariate Logistic regression was used to determine independent predictors and construct the model. More importantly, not only the discrimination (AUC) of the model was concerned, but also its calibration was comprehensively evaluated through calibration curves and the H-L test, and its clinical utility was proven through DCA. The verification of these three aspects provides a solid guarantee for the real-world application of the model.

The clinical significance of this study lies in that the nomogram model transforms the complex prediction equation into an easy-to-use clinical tool in a visual form. At the bedside, doctors can quickly estimate the adverse risk of patients based on the five easily obtainable indicators at the initial stage after admission to the ICU. This is crucial for the early identification of the patient group who “seem stable but are actually at high risk”. For these high-risk patients identified by the model, this study provides a rationale for exploring targeted intestinal barrier protection strategies (such as precise hemodynamic management to avoid intestinal ischemia, early enteral nutrition, and the use of potential intestinal mucosal protectants) in future clinical practice. However, whether such strategies can block the vicious cycle of enterogenic sepsis deterioration and improve prognosis requires verification through well-designed clinical trials.

However, this study also has several limitations. First, this is a single-center study. Although the internal validation results are good, the external universality of the model still needs to be further verified through multi-center, large-sample prospective studies. Second, the biomarkers were only detected at a single time point within 24 h after admission to the ICU, and the relationship between their dynamic change trajectories and the prognosis was not observed. Future studies can include sequential measurements at multiple time points to explore their dynamic predictive value. Third, the total sample size (352 cases) may be relatively small for a multivariable model with five predictors, which may restrict the model's generalizability despite controllable overfitting risk (verified by bootstrapping validation). Fourth, the inclusion criterion requiring available I-FABP and D-lactate measurements may exclude some typical sepsis patients (who did not undergo these tests in clinical practice), introducing selection bias and restricting the model's applicability to patients with such biomarker data. Fifth, although the model includes key indicators, there are still other potentially important factors (such as micro biomics and immunological indicators) not included. Sixth, the selected 28-day composite poor prognosis outcome lacks validation against direct gold standards for intestinal barrier function (e.g., sugar absorption tests, endoscopy), and classifying this outcome as “intestinal barrier dysfunction” overstates the link between the outcome and intestinal status. Seventh, the model only incorporates two intestinal barrier surrogate biomarkers (I-FABP, D-lactate) (not direct markers of barrier integrity) and lacks validation against gold standards for intestinal barrier function (e.g., sugar permeability tests, histopathology). Thus, it cannot be used to directly evaluate intestinal barrier function and should be distinguished from direct barrier assessment tools. Eighth, the model omits several clinically essential confounders, including microbiological etiology (bacterial, fungal, viral, or mixed infection), source of infection (pulmonary, abdominal, etc.), key therapeutic factors (timing of antibiotics, adequacy of source control), and prior gastrointestinal diseases. These factors may influence both intestinal biomarker levels and patient prognosis, leading to residual bias as they were not adjusted for. Ninth, although the model includes key indicators, there are still other potentially important factors (such as micro biomics and immunological indicators) not included. Additionally, non-intestinal confounding factors (e.g., underlying cardiopulmonary disease, infection source) may contribute to the need for life support, introducing bias into the inference of intestinal barrier-related outcomes. Future studies should adjust for these confounders via multivariate analysis.

## Conclusion

This study successfully constructed and internally validated a nomogram prediction model integrating intestinal barrier surrogate biomarkers (I-FABP, D-lactate) and systemic clinical indicators (admission SOFA score, blood lactate, PCT) for 28-day composite poor prognosis prediction in severe sepsis. The model showed moderate-to-good discriminatory ability in the training set (AUC = 0.771, C-index = 0.771) and moderate discriminatory ability in the validation set (AUC = 0.641, C-index = 0.641), with acceptable calibration (Hosmer-Lemeshow test *P* > 0.05) and clinical utility confirmed by decision curve analysis.

This model provides a preliminary decision-support tool for clinicians to conduct early risk stratification of severe sepsis patients, especially in identifying those at high risk of intestinal barrier-related poor prognosis. However, the study has several limitations: lack of external multi-center validation, use of indirect intestinal barrier biomarkers without gold standard verification, and omission of key confounders such as infection source and therapeutic factors. Future studies should address these limitations through multi-center prospective designs, integration of direct intestinal barrier assessment methods, and inclusion of additional confounders to optimize the model.

Overall, this study highlights the potential value of integrating intestinal barrier biomarkers into sepsis prognosis prediction and provides a foundation for subsequent clinical translation and model optimization.

## Data Availability

The original contributions presented in the study are included in the article/Supplementary material, further inquiries can be directed to the corresponding author.
