# Supplementary material for: Construction and validation of a nomogram based on clinical indicators for 28-day composite poor prognosis prediction in severe sepsis
Source: Front Public Health. 2026 Feb 10;14:1709504. doi: 10.3389/fpubh.2026.1709504 (PMC12929523; doi:10.3389/fpubh.2026.1709504)
Supplement: Supplementary file 1 [file Table_1.docx]

**Supplemental Table 1** The results of bootstrapping (1000 repetitions) and 5-fold cross-validation

| Validation Method | Group | C-index (95% CI) | Mean AUC (95% CI) |
| --- | --- | --- | --- |
| Bootstrapping (1000 repetitions) | Training set | 0.753 (0.680–0.826) | 0.753 (0.680–0.826) |
| Bootstrapping (1000 repetitions) | Validation set | 0.628 (0.501–0.755) | 0.628 (0.501–0.755) |
| 5-fold cross-validation | Training set | 0.732 (0.681–0.783) | 0.732 (0.681–0.783) |
| 5-fold cross-validation | Validation set | 0.615 (0.502–0.728) | 0.615 (0.502–0.728) |
