# Supplementary material for: Construction and validation of a nomogram based on clinical indicators for 28-day composite poor prognosis prediction in severe sepsis
Source: Front Public Health. 2026 Feb 10;14:1709504. doi: 10.3389/fpubh.2026.1709504 (PMC12929523; doi:10.3389/fpubh.2026.1709504)
Supplement: Supplementary file 2 [file Supplementary_file_1.docx]

**
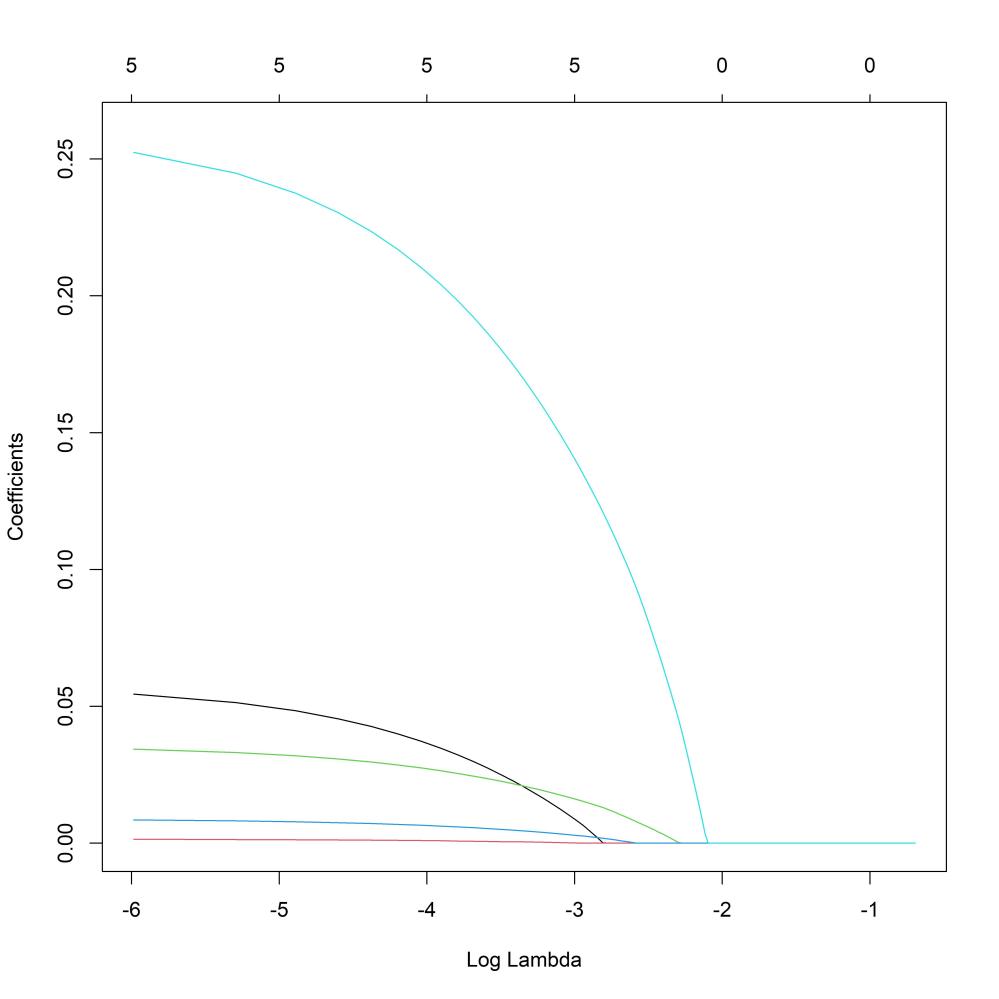
**

**Supplemental Figure 1.** LASSO Regression Analysis Diagram

**
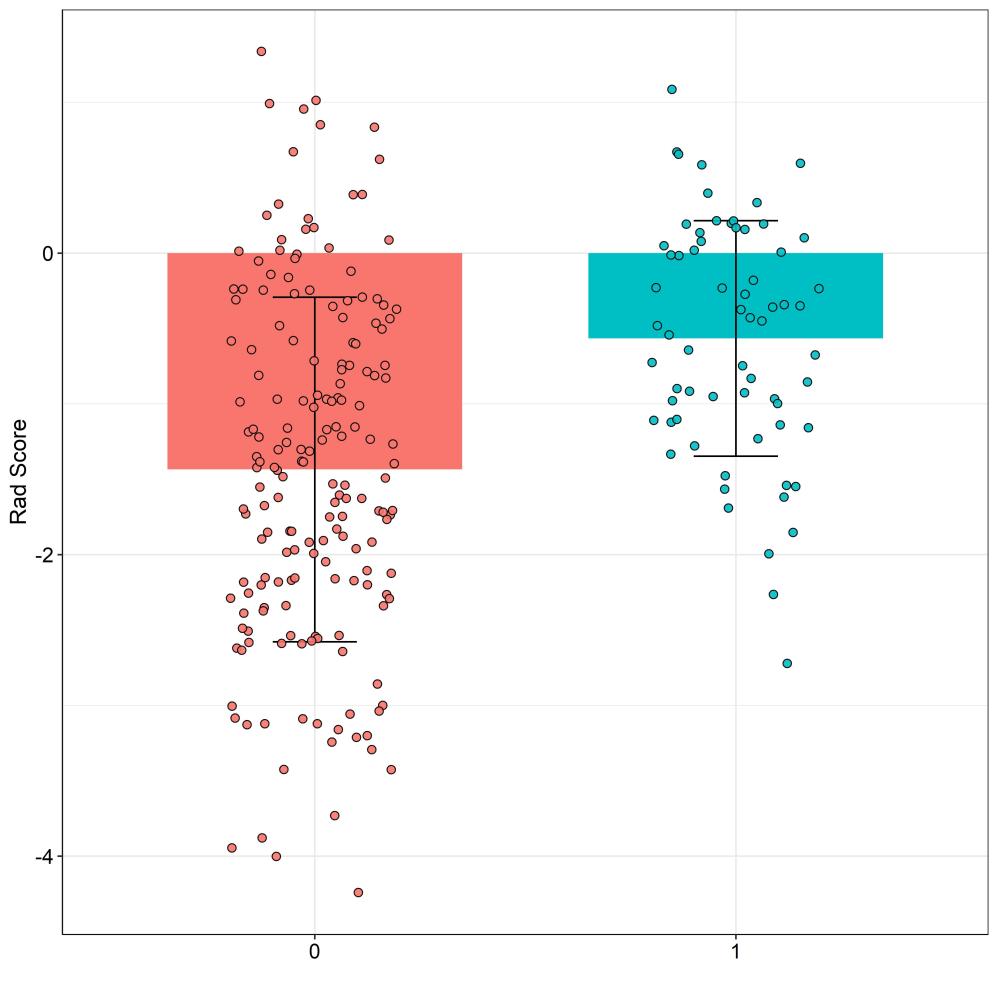
**

**Supplemental Figure 2.** Comparison of LASSO Score Differences


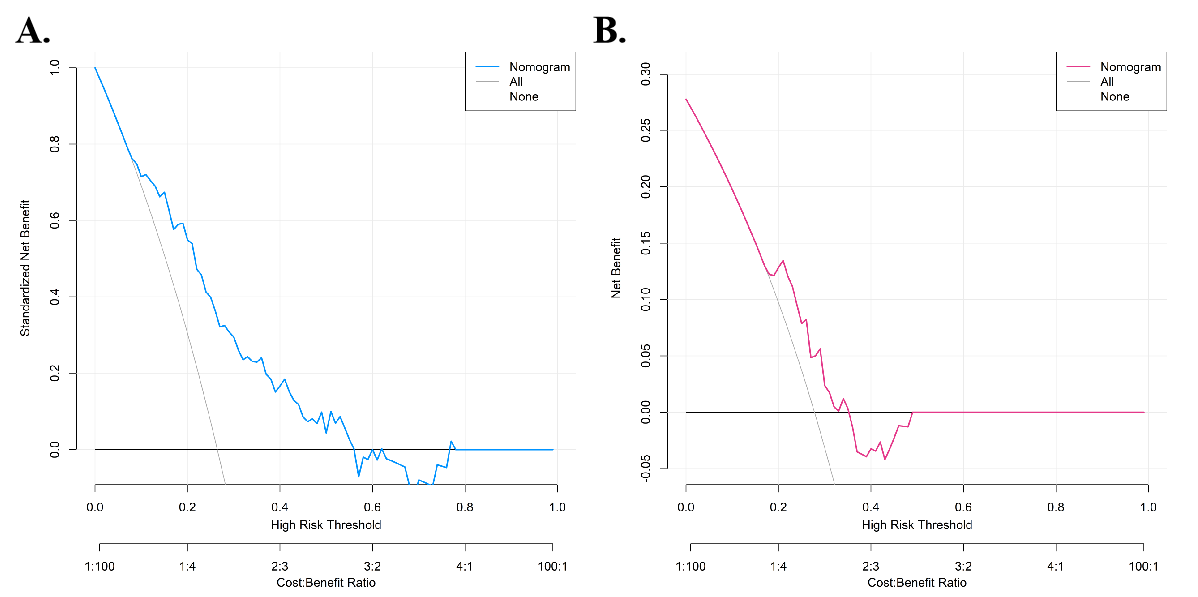


**Supplemental Figure 3.** Decision curves in the training set (A) and the validation set (B)
